# Supplementary material for: Differentiating Drosophila female germ cells initiate Polycomb silencing by regulating PRC2-interacting proteins
Source: eLife. 2020 Aug 10;9:e56922. doi: 10.7554/eLife.56922 (PMC7438113; doi:10.7554/eLife.56922)
Supplement: Transparent reporting form [file elife-56922-transrepform.docx]

***eLife’s* transparent reporting form**

We encourage authors to provide detailed information *within their submission* to facilitate the interpretation and replication of experiments. Authors can upload supporting documentation to indicate the use of appropriate reporting guidelines for health-related research (see [EQUATOR Network](http://www.equator-network.org/%20)), life science research (see the [BioSharing Information Resource](https://biosharing.org/)), or the [ARRIVE guidelines](http://www.plosbiology.org/article/info:doi/10.1371/journal.pbio.1000412) for reporting work involving animal research. Where applicable, authors should refer to any relevant reporting standards documents in this form.

If you have any questions, please consult our Journal Policies and/or contact us: [editorial@elifesciences.org](mailto:editorial@elifesciences.org).

**Sample-size estimation**

- You should state whether an appropriate sample size was computed when the study was being designed
- You should state the statistical method of sample size computation and any required assumptions
- If no explicit power analysis was used, you should describe how you decided what sample (replicate) size (number) to use

Please outline where this information can be found within the submission (e.g., sections or figure legends), or explain why this information doesn’t apply to your submission:

Sample sizes were determined based on experience with similar experiments to set minimums as well as cost and diminishing returns to set maximums. This information is provided in Figure Legends or Methods. Generally, images of germaria, ovarioles or ovarian follicles are representative of more than 3 replicates from at least 3 separate females. At least 3 ovary pairs were analyzed for each hsGFP reporter assay or antibody staining as described in Methods. We repeated each experiment on different days (technical replicates) but present experiments done in parallel (on the same day) to minimize technical variation and highlight biological variation.

**Replicates**

- You should report how often each experiment was performed
- You should include a definition of biological versus technical replication
- The data obtained should be provided and sufficient information should be provided to indicate the number of independent biological and/or technical replicates
- If you encountered any outliers, you should describe how these were handled
- Criteria for exclusion/inclusion of data should be clearly stated
- High-throughput sequence data should be uploaded before submission, with a private link for reviewers provided (these are available from both GEO and ArrayExpress)

Please outline where this information can be found within the submission (e.g., sections or figure legends), or explain why this information doesn’t apply to your submission:

A **biological replicate** is an experiment performed using separately derived biological starting material. This would typically be ovarian tissue obtained from females of identical genotype, but derived and analyzed in separate experiments. A **technical replicate** is an experimental analysis of inanimate material or an independent dataset performed separately, usually on a different day. The number of replicates is given in Figure Legends or the Methods. We did not exclude any outliers. All data is available from GSE145282. All code was submitted to GitHub (see Methods).

**Statistical reporting**

- Statistical analysis methods should be described and justified
- Raw data should be presented in figures whenever informative to do so (typically when N per group is less than 10)
- For each experiment, you should identify the statistical tests used, exact values of N, definitions of center, methods of multiple test correction, and dispersion and precision measures (e.g., mean, median, SD, SEM, confidence intervals; and, for the major substantive results, a measure of effect size (e.g., Pearson's r, Cohen's d)
- Report exact p-values wherever possible alongside the summary statistics and 95% confidence intervals. These should be reported for all key questions and not only when the p-value is less than 0.05.

Please outline where this information can be found within the submission (e.g., sections or figure legends), or explain why this information doesn’t apply to your submission:

Statistical methods used are reported in the Figure Legends. For example, in Fig. 1H, the mean +/- 1 standard deviation at each point is shaded. Expression was taken as different at the developmental time when the shaded regions of the experimental sample and EZ control no longer overlapped and for as long as they remained separate.

(For large datasets, or papers with a very large number of statistical tests, you may upload a single table file with tests, Ns, etc., with reference to sections in the manuscript.)

**Group allocation**

- Indicate how samples were allocated into experimental groups (in the case of clinical studies, please specify allocation to treatment method); if randomization was used, please also state if restricted randomization was applied
- Indicate if masking was used during group allocation, data collection and/or data analysis

Please outline where this information can be found within the submission (e.g., sections or figure legends), or explain why this information doesn’t apply to your submission:

Groups were allocated according to genotype and conditions as described for each experiment. Masking in ChIPseq analysis is described in methods.

**Additional data files (“source data”)**

- We encourage you to upload relevant additional data files, such as numerical data that are represented as a graph in a figure, or as a summary table
- Where provided, these should be in the most useful format, and they can be uploaded as “Source data” files linked to a main figure or table
- Include model definition files including the full list of parameters used
- Include code used for data analysis (e.g., R, MatLab)
- Avoid stating that data files are “available upon request”

Please indicate the figures or tables for which source data files have been provided:

1) Raw mean GFP fluorescence intensity measured with ImageJ for reporter experiments in Figure 1G-H and Figure 6A.

2) Raw mean GFP fluorescence intensity measured with ImageJ for reporter experiments in Figure 6C.

3) Raw mean antibody fluorescence intensity measured with ImageJ for H3K27me staining in Figure 6B.

4) Raw fastq files for all RNAseq and ChIPseq experiments (GEO)

5) Table of gene TPMs from Stringtie in all RNAseq samples (GEO)

6) Raw Read Counts for all genes from all RNAseq samples used for DESeq2 (GEO)

7) Normalized BigWig coverage files for all ChIPseq samples (GEO)

8) Read depth in 5kb bins tiling the genome used in figures 2 and 5 (GEO)
